# Bilateral Low-Frequency Air–Bone Gap Following Spinal Anesthesia: An Unusual Audiometric Presentation: Case Report

**DOI:** 10.3390/reports9030212

**Published:** 2026-07-04

**Authors:** Konstantina Dinaki, Rafail Ioannidis, Panagiotis Theodorou, Aristidis Delis, Constantinos Papadopoulos

**Affiliations:** 1 Department of ENT Surgery, General Hospital of Mytilene Vostaneio, 81132 Lesvos, Greece; konnidin@windowslive.com (K.D.);; 2 Department of Anesthesiology and Pain Medicine, General Hospital of Drama, 66100 Drama, Greece; raphaioan@gmail.com; 3 Department of ENT Surgery, General Hospital of Drama, 66100 Drama, Greece

**Keywords:** hearing loss, bilateral, spinal anesthesia

## Abstract

**Background and Clinical Significance:** To report an unusual case of a bilateral low-frequency air–bone gap consistent with an apparent conductive audiometric pattern following spinal anesthesia and discuss a possible underlying mechanism; **Case Presentation:** A 56-year-old man underwent elective inguinal hernia repair under spinal anesthesia. On the second postoperative day, he developed a severe postural headache followed by bilateral hearing loss. Otoscopic examination was normal. Tuning fork tests and pure-tone audiometry demonstrated a bilateral low-frequency air–bone gap consistent with an apparent conductive audiometric pattern. Laboratory findings were unremarkable. The patient was managed conservatively with bed rest, hydration and systemic corticosteroids, resulting in gradual clinical improvement; **Conclusions:** Hearing loss after spinal anesthesia is typically sensorineural and attributed to cerebrospinal fluid pressure alterations. This case highlights a rare apparent conductive audiometric pattern in the absence of clinically evident middle-ear pathology. A possible mechanism may involve altered inner-ear pressure dynamics leading to transient mechanical restriction of stapes mobility. Awareness of this atypical presentation may facilitate prompt recognition and appropriate management.

## 1. Introduction and Clinical Significance

Perioperative hearing loss (POHL) is an uncommon but clinically significant complication associated with anesthesia. It has been reported more frequently following neuraxial techniques, particularly spinal anesthesia, than after general anesthesia [1]. However, the true incidence of POHL remains uncertain, as it is likely underdiagnosed due to its often-subclinical presentation and the lack of routine postoperative audiometric assessment [2]. In the majority of cases, hearing impairment is transient and resolves spontaneously within a few days without the need for specific therapeutic intervention [3].

The pathophysiology of POHL following subarachnoid anesthesia is not yet fully elucidated, but it is most commonly attributed to cerebrospinal fluid (CSF) leakage and the subsequent reduction in intracranial pressure. This decrease in pressure may be transmitted to the inner ear via the cochlear aqueduct, resulting in a reduction in perilymphatic pressure [2,4,5]. The resulting imbalance between perilymphatic and endolymphatic pressures may lead to endolymphatic hydrops, displacement of the basilar membrane, and subsequent auditory dysfunction, typically affecting low-frequency hearing [3,4].

Most reported cases of POHL describe sensorineural hearing loss, supporting the hypothesis that inner ear pressure alterations play a central role in its development. Conductive hearing loss in this setting is exceedingly rare and poorly understood, with limited documentation in the literature. This highlights an important gap in current knowledge regarding the full spectrum of auditory complications associated with spinal anesthesia. Early recognition of such atypical presentations is important for accurate diagnosis, appropriate management, and avoidance of unnecessary interventions.

In this context, we present a unique case of a bilateral low-frequency air–bone gap consistent with an apparent conductive audiometric pattern following spinal anesthesia for inguinal hernia repair. A review of the available literature identified multiple reports of sensorineural hearing loss following spinal anesthesia; however, we found no published reports describing bilateral conductive hearing loss with a documented low-frequency air–bone gap after spinal anesthesia. To the best of our knowledge, this represents the first such case.

## 2. Case Presentation

A 56-year-old man with no significant past medical history, including no previous surgeries, underwent elective inguinal hernia repair under spinal anesthesia. Following prehydration with 500 mL of lactated Ringer’s solution, spinal anesthesia was performed with the patient in the sitting position at the L3–L4 intervertebral space using a midline approach. A 27-gauge atraumatic pencil-point spinal needle was inserted. After aseptic preparation of the puncture site, and after confirmation of free flow of clear cerebrospinal fluid, 12.5 mg of hyperbaric bupivacaine combined with 15 μg of fentanyl was administered intrathecally. The procedure was completed uneventfully on the first attempt. A sensory block to the T6 dermatome was achieved, with a Bromage score of 3. During surgery, an additional 500 mL of lactated Ringer’s solution was administered intravenously. Hemodynamic parameters remained stable throughout the perioperative period. The patient was discharged 24 h postoperatively.

On the second postoperative day, the patient developed a severe headache with postural characteristics. The following day, he reported bilateral hearing loss and was referred to the ENT Department. He denied auditory fullness, otalgia, vertigo, or tinnitus. Otoscopic examination was unremarkable. Tympanometry demonstrated bilateral Type A tympanograms, indicating normal middle-ear pressure and tympanic membrane mobility. The patient reported no previous hearing impairment, otologic symptoms, or history of ear disease before surgery. Bedside tuning fork tests (Weber and Rinne) suggested bilateral conductive hearing loss.

**Figure 1:**
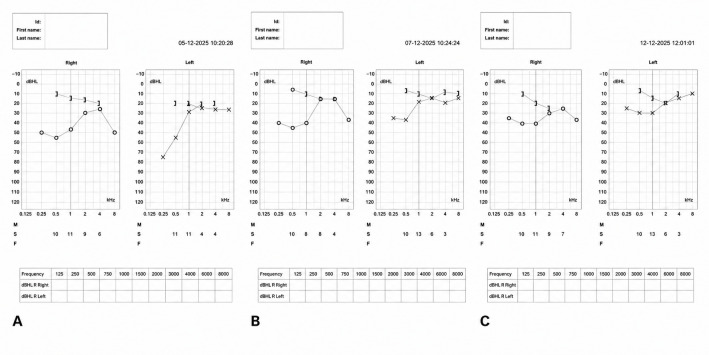
(**A**): Baseline pure-tone audiogram demonstrating a bilateral low-frequency air–bone gap consistent with an apparent conductive audiometric pattern at symptom onset. (**B**,**C**): Follow-up pure-tone audiogram on second and seventh day of conservative treatment showing significant improvement in air conduction thresholds.

**Figure 2:**
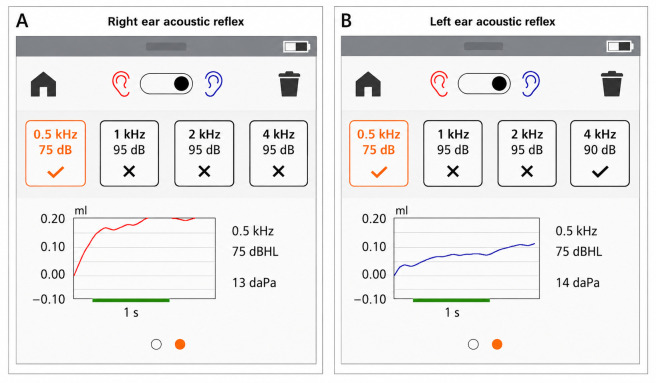
Stapedius acoustic reflex testing. (**A**) Right ear: ipsilateral reflex present at 0.5 kHz (75 dB HL) and absent at 1, 2 and 4 kHz (up to 95 dB HL). (**B**) Left ear: ipsilateral reflex present at 0.5 kHz (75 dB HL) and absent at 1 and 2 kHz (up to 95 dB HL) and at 4 kHz (up to 90 dB HL).

Pure-tone audiometry demonstrated a bilateral low-frequency air–bone gap consistent with an apparent conductive audiometric pattern (Figure 1A). A substantial air–bone gap was observed at the lower frequencies (250–500 Hz) bilaterally, with progressive reduction toward the higher frequencies. Audiometry was performed according to standard clinical procedures, and the audiometric configuration was reproducible on subsequent examinations, making a masking artifact unlikely. Acoustic stapedius reflex testing revealed preserved ipsilateral reflexes at 0.5 kHz at 75 dB HL, whereas ipsilateral reflexes were absent at 1, 2, and 4 kHz bilaterally, even at stimulus intensities up to 95 dB HL (Figure 2). Laboratory investigations were within normal limits.

Conservative treatment consisting of bed rest in the supine position, adequate oral and intravenous hydration, and systemic corticosteroids was initiated on postoperative day 3, immediately following audiological evaluation. Clinical improvement was noted two days after treatment initiation and was confirmed by repeat audiometry. A follow-up audiogram obtained seven days after treatment initiation demonstrated near-complete recovery of hearing thresholds (Figure 1). The patient was discharged after seven days of hospitalization and continued oral corticosteroid therapy on an outpatient basis. Systemic corticosteroids were administered empirically because hearing loss following spinal anesthesia is most commonly reported as sensorineural hearing loss, a condition for which corticosteroids are frequently used in clinical practice. However, the contribution of corticosteroid therapy to the patient’s recovery cannot be determined from this case.

## 3. Discussion

Hearing loss following spinal anesthesia has been previously documented; however, its incidence is likely underestimated. The vast majority of reported cases describe transient sensorineural hearing loss, typically resolving spontaneously within 5–15 days and predominantly affecting low frequencies (125–1000 Hz) [6,7]. The auditory function of the inner ear is closely related to cerebrospinal fluid dynamics. Perilymph, which surrounds the membranous labyrinth, is partially derived from CSF, and alterations in CSF pressure can directly affect cochlear mechanics. Endolymph is contained within the membranous labyrinth. Ions are transported between the endolymph and perilymph through passive diffusion and active transport [2].

Following dural puncture, CSF leakage may lead to intracranial and perilymphatic pressure reduction. This pressure alteration is transmitted to the inner ear, resulting in an imbalance between perilymph and endolymph [2]. Such imbalance may lead to endolymphatic hydrops, displacement of the basilar membrane and subsequent hearing impairment, most commonly of the sensorineural type [4,8].

In contrast to the predominantly reported sensorineural hearing loss, our patient demonstrated a clear bilateral conductive audiometric pattern, which, to our knowledge, has not been previously reported. Conductive hearing loss is defined by the presence of an air–bone gap (ABG) on pure-tone audiometry, with a difference of at least 15 dB HL between air and bone conduction thresholds at a given frequency. Typically, this pattern is associated with pathology of the external or middle ear, such as cerumen impaction, middle ear effusion, tympanic membrane perforation, or otosclerosis [9]. However, in our case, otoscopic examination and clinical evaluation revealed no evidence of middle ear disease.

Unexplained conductive hearing loss in the absence of middle ear disease has been described in association with several inner ear disorders, including enlarged vestibular aqueduct, superior semicircular canal dehiscence and perilymphatic fistula [9,10]. In such cases, the air–bone gap is often attributed to a “third-window” effect, resulting in dissipation of acoustic energy [9].

A possible explanation in our case is that CSF hypotension may alter perilymphatic pressure, leading to secondary endolymphatic imbalance. Increased endolymphatic volume may exert pressure on the medial surface of the stapes footplate, reducing its mobility and producing an apparent conductive hearing loss despite the absence of middle ear pathology. As cochlear fluids are incompressible, this increased pressure may restrict stapedial mobility, mimicking a conductive hearing loss on audiometry [9,,11]. An alternative mechanism may involve saccular dilatation causing direct mechanical interaction with the stapes footplate [11,12]. Previous gadolinium-enhanced imaging studies in patients with endolymphatic hydrops have suggested that direct contact between a distended saccule and the stapes footplate may contribute to the development of an air–bone gap [9,11].

In our patient, the temporal association between spinal anesthesia, post-dural puncture headache and the onset of hearing loss supports CSF pressure alteration as the initiating event. The frequency-dependent preservation of acoustic reflexes, limited to low frequencies, parallels the low-frequency air–bone gap and may support a non–middle ear mechanism. Although this observation is intriguing, it should be interpreted cautiously given the single-case nature of this report. The absence of reflexes at higher frequencies does not contradict this interpretation, as reflex thresholds may be more susceptible to subtle inner ear mechanical changes.

The favourable clinical course, with gradual resolution following conservative management including bed rest, hydration and corticosteroid therapy, is consistent with the transient nature of inner ear pressure disturbances. The temporal association with postural headache further supports cerebrospinal fluid hypotension as the initiating event.

This case highlights an unusual audiological presentation following spinal anesthesia and expands the spectrum of hearing complications associated with CSF pressure alterations. A focused review of the literature identified only sensorineural or mixed hearing loss patterns following spinal anesthesia, while a purely bilateral conductive audiometric presentation was not found.

Alternative explanations should also be considered. Endolymphatic hydrops, Ménière-like inner-ear dysfunction, retrocochlear pathology, and pre-existing conductive disorders represent potential differential diagnoses. However, the absence of vertigo, tinnitus, aural fullness, neurological symptoms, and the documented temporal association with post-dural puncture headache made these explanations less likely in the present case. Nevertheless, the absence of advanced imaging precludes definitive exclusion of these entities.

Several limitations should be acknowledged. Preoperative audiometric data were unavailable; therefore, a pre-existing conductive hearing loss cannot be definitively excluded. Furthermore, temporal bone CT or MRI imaging was not performed; consequently, otosclerosis, third-window lesions, and other structural causes of conductive hearing loss cannot be completely ruled out. These limitations should be considered when interpreting the proposed pathophysiological mechanism.

## 4. Conclusions

A bilateral low-frequency air–bone gap with an apparent conductive audiometric pattern following spinal anesthesia appears to be an exceptionally rare complication. This case raises the possibility that alterations in cerebrospinal fluid pressure may, in rare instances, lead to a reversible low-frequency air–bone gap with an apparent conductive audiometric pattern in the absence of middle ear pathology, possibly through mechanisms related to endolymphatic hydrops and impaired stapes mobility. Awareness of this atypical presentation is important for early recognition, appropriate management, and reassurance of affected patients. In the present case, conservative treatment was associated with a favorable clinical outcome.

## Data Availability

The original contributions presented in this study are included in the article. Further inquiries can be directed to the corresponding author.

## Abbreviations

The following abbreviations are used in this manuscript:

POHL	Perioperative hearing loss
CSF	Cerebrospinal fluid
ABG	Air–bone gap
